# A Study on the Aging Mechanism and Anti-Aging Properties of Nitrile Butadiene Rubber: Experimental Characterization and Molecular Simulation

**DOI:** 10.3390/polym17111446

**Published:** 2025-05-23

**Authors:** Min Zhu, Hanyuan Huang, Haiyan Li, Gui Huang, Jingjing Lan, Jing Fu, Juqin Fan, Yujun Liu, Zhiwu Ke, Xiaojie Guo, Hongkuan Zhou, Yan Li

**Affiliations:** 1 Naval University of Engineering, Wuhan 430033, China; min0zhu@163.com (M.Z.); h20209999@163.com (H.H.); tangerine1@163.com (J.L.); fujing_2005@sina.com (J.F.); 15172515814@163.com (J.F.); lyj2424@163.com (Y.L.); yacareft@163.com (Y.L.); 2 China State Shipbuilding Corporation, Limited (CSSC) 719th Research Institute, Wuhan 430205, China; keylab_rndl@163.com (Z.K.); lucky_gxj_0372@163.com (X.G.); hongkuanzhou@hust.edu.cn (H.Z.)

**Keywords:** rubber seal, aging, molecular model

## Abstract

To tackle the degradation of sealing performance in nitrile butadiene rubber (NBR) seals due to material aging during long-term service, this study integrates experimental and molecular simulation methods to elucidate the aging mechanism. Experimental results reveal that the contents of C=C and C=O functional groups significantly decrease during aging, accompanied by enhanced hydrophobicity and increased crosslink density of NBR, indicating that crosslinking reactions dominate the aging process with the participation of C=C and C=O groups. Quantum mechanics (QM) and molecular dynamics (MD) simulations further demonstrate that α-H, C=C, and C≡N groups are preferentially oxidized due to their low bond energies. The oxidation of NBR generates unstable epoxy intermediates, which undergo chain scission to form ketones, aldehydes, and ultimately crosslinked structures. Using a multi-dimensional evaluation system based on bond dissociation energy (BDE), solubility parameter (Δδ), and migration coefficient (MSD), four antioxidants (4010NA, 4010, MC, and BHT) were screened. BHT emerges as the optimal choice, exhibiting superior free radical scavenging ability (BDE = 346.3 kJ/mol), good matrix compatibility (Δδ = 2.95), and anti-migration properties. The MD-based screening method established herein provides a theoretical basis for designing antioxidant systems in high-performance rubber materials, facilitating the development of advanced rubber products.

## 1. Introduction

Nitrile butadiene rubber is widely used as a sealing ring in mechanical equipment due to its excellent wear resistance, airtightness, and waterproof performance. During long-term operation under humid, high-temperature, and compressive conditions, seals experience stress relaxation and compression set, leading to reduced contact stress with clamping rings and gradual loss of sealing efficiency. Therefore, investigating the aging mechanism of NBR seals is critical for ensuring equipment safety.

Analyzing failure mechanisms serves as the foundation for describing and confirming material degradation. Current research has established a multi-scale characterization system to dissect this process: aging-induced mechanical property degradation is accelerated under humid–heat conditions, with compression set rates significantly higher than those in dry environments [1]. NBR aging damage is severe and non-uniform, with the most pronounced degradation occurring at edge regions. Crosslinking dominates the aging process [2,3], during which tensile strength initially increases with crosslink density but declines after reaching a peak [3]. FTIR and X-ray photoelectron spectroscopy (XPS) reveal the hydrolysis of nitrile groups during aging, accompanied by the formation of hydroxyl and carbonyl groups [4,5,6]. FTIR results also indicate plasticizer migration to the rubber surface during thermo-oxidative aging, with a competitive interplay between plasticizer volatilization and medium diffusion in oil environments—an effect more pronounced than in hot air [7].

While experimental studies have significantly advanced aging mechanism exploration, challenges remain: aging tests are time-consuming, and complex degradation pathways are difficult to characterize [8]. With breakthroughs in computational chemistry, multi-scale simulation techniques have emerged as a new paradigm for unraveling rubber aging mechanisms. At the microscale, quantum mechanics (QM) simulations are widely used to quantitatively analyze reaction energetics, such as transition state barriers and reaction free energies, and to derive thermodynamic parameters for hydrogen bonding interactions [9,10].

To enhance the antioxidant capacity of rubber composites, antioxidants are typically incorporated. Selection criteria involve analyzing their interactions with rubber matrices, with priority given to matrix compatibility—a parameter directly impacting composite mechanical properties—and key factors such as radical scavenging activity and migration resistance [11]. Traditional antioxidant screening for rubber composites requires extensive experimental work, whereas molecular dynamics (MD) simulations enable rapid prescreening by calculating solubility parameters (δ) to assess additive-matrix compatibility [12] and evaluating migration resistance through metrics like Mean square displacement (MSD) and free volume fraction [13].

**Figure 1:**
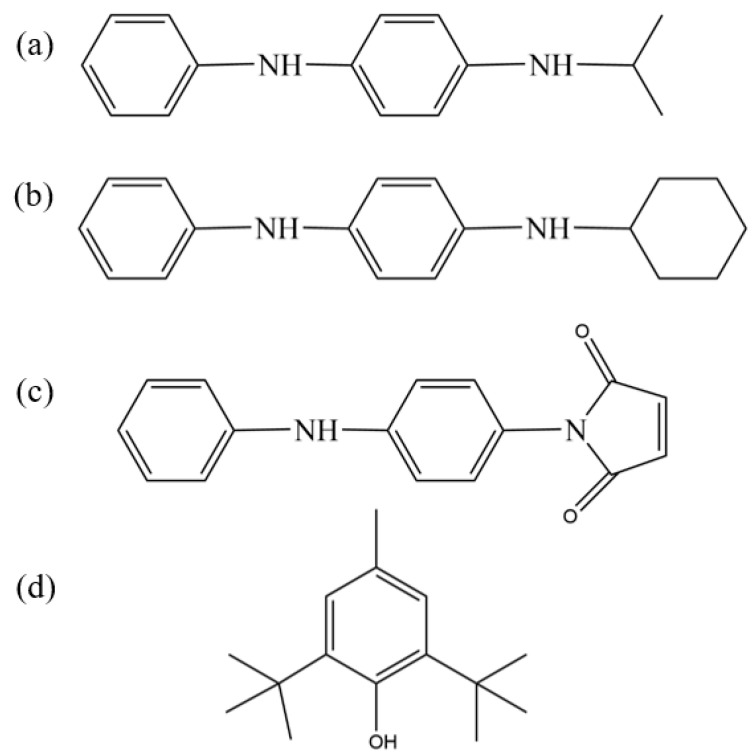
Chemical structures of antioxidants: (**a**) 4010NA; (**b**) 4010; (**c**) MC; and (**d**) BHT.

In this study, we comprehensively evaluated the antioxidant capabilities and compatibility with nitrile–butadiene rubber (NBR) of four commercially available antioxidants, amine-type antioxidants 4010NA (N-isopropyl-N′-phenyl-p-phenylenediamine) and 4010 (N-cyclohexyl-N′-phenyl-p-phenylenediamine), phenolic antioxidant BHT (2,6-di-tert-butyl-p-cresol), and reactive antioxidant MC (N-(4-anilinophenyl)maleimide), whose molecular structures are presented in Figure 1. The amine-type antioxidants demonstrated strong free-radical scavenging abilities, making them particularly suitable for high-temperature environments; however, their discoloration and contamination tendencies limit their application mainly to dark-colored or black rubber and plastic products [14]. In contrast, the phenolic antioxidant BHT relied on its phenolic hydroxyl group to provide effective antioxidant protection at medium and low temperatures but suffered from limitations such as relatively low polarity matching degree, limited compatibility, and insufficient high-temperature stability [15]. Notably, the reactive antioxidant MC exhibited a unique dual functionality by covalently bonding to the rubber matrix or filler surface through chemical grafting of its double bond and aniline group, thereby achieving both “permanent” antioxidant properties and improved filler–rubber compatibility, which significantly inhibited antioxidant migration and enhanced interfacial bonding strength [16]. These works collectively provide valuable insights and guidance for the development of high-performance nitrile–butadiene rubber formulations.

In this study, we simulated the service environment of NBR and conducted thermo-mechanical accelerated aging tests to investigate surface property and crosslink density changes using FTIR, crosslink density analysis, and contact angle measurements. MD simulations were employed to analyze bond energies in NBR’s typical chemical structures, identify degradation-prone sites during aging, and elucidate the underlying aging mechanisms. Four antioxidants—4010NA, 4010, MC, and BHT—were systematically evaluated for their compatibility, antioxidant efficiency, and migration resistance in NBR matrices. This work provides theoretical guidance for developing polymer composites with optimized anti-aging and mechanical properties through multi-scale analysis of antioxidant-NBR interactions.

## 2. Methods

### 2.1. Aging of the Sample

The NBR used in this study was provided by Qingdao Yike Rubber & Plastics Co., Ltd. in Qingdao, China (specific formulation details were withheld by the company for commercial confidentiality). Test specimens were prepared according to GB/T 1683 standards [17] by cutting the NBR into small cylinders with dimensions of φ 10 mm × 10 mm (±0.2 mm height). Accelerated aging tests were conducted under a 30% compression ratio to simulate the actual service conditions of NBR seals. The aging procedure was conducted in a blast drying oven (HW-490 high-temperature aging chamber, manufactured by Aerospace Reliance Corporation in Tianjing, China). The aging temperature was set at 80 °C, which, based on our prior experimental results, exhibits a failure mechanism consistent with that at ambient temperature while enabling a moderate aging rate [18]. Specimens were extracted for characterization after aging at 80 °C under a 30% compression ratio for durations of 19 h, 76 h, 152 h, and 228 h, respectively.

### 2.2. Performance Test

Attenuated Total Reflection–Fourier Transform Infrared Spectroscopy (ATR-FTIR): Surface functional group changes during NBR aging were monitored via ATR-FTIR. A Nicolet 80 spectrometer (Thermo Fisher Scientific, Waltham, MA, USA) was used with a wavenumber range of 4000–500 cm^−1^, a resolution of 4 cm^−1^, and 32 co-added scans to reduce noise.

The sessile drop method using an SL200 contact angle analyzer (KINO, New York, NY, USA) was executed under controlled conditions maintained by HVAC systems: droplet volume = 2.0 ± 0.1 μL (0.5 mm needle), T = 25 ± 0.5 °C, and RH = 50–60%. Three independent measurements were taken at distinct regions (edge, center, and transition zone) per sample, with outliers excluded prior to mean calculation.

Crosslink Density Test: Crosslink density evolution during aging was measured using a modified equilibrium swelling method. Sulfured rubber samples (0.12–0.13 g, m_0_) were swollen in a solution of 20 mL toluene and 5 mL ammonia (28 wt%) for 48 h at room temperature. Here, the role of ammonia water is to break the hydrogen bond interaction between the macromolecular chains and the fillers, ensuring that the obtained crosslinking density is derived more from the chemical crosslinking of the molecular chains. Swollen samples were dried to constant mass (m_2_) in a vacuum oven at 70 °C. Crosslink density was calculated using the Flory–Rehner equation [19,20]. Three replicates per condition were averaged.

(1) $$\upsilon_{e}=-\frac{\ln(1-\upsilon_{f})+\upsilon_{f}+\chi \upsilon_{f}^{2}}{V_{S}(\upsilon_{f}^{1/3}-\upsilon_{f}/2)}$$

where *V_S_* represents the molar volume of toluene (106.5 mL/mol), *χ* denotes the Flory–Huggins interaction parameter between the polymer and toluene (0.435) [16], and *ν_f_* is the volume fraction of the polymer at equilibrium swelling, which is calculated using Equation (2):

(2) $$\upsilon_{f}=\frac{m_{2}/\rho_{p}}{m_{2}/\rho_{p}+(m_{1}-m_{2})/\rho_{s}}$$

where *ρ_p_* is the density of the NBR (1.02 g/cm^3^) and *ρ_s_* is the density of toluene (0.86 g/cm^3^).

### 2.3. Molecular Dynamics Simulation

Bond Dissociation Energy (BDE) Calculation: The Materials Studio (MS) 2020 software (20.1 X64) was used to construct NBR models, and bond dissociation energies of NBR’s typical chemical structures were calculated using the Dmol^3^ module. The BDE calculation formula is as follows:

(3) ΔG=E(A•)+E(B•)−E(AB)

where *E*(*A*), *E*(*B*), and *E*(*AB*) denote the energies of cleavage products *A*, *B*, and the reactant molecule *AB*, respectively.

MD simulations were performed using MS to construct unit cells for pure NBR, 4010NA, 4010, MC, and BHT. Solubility parameters of these systems were analyzed, followed by the creation of NBR/4010NA, NBR/4010, NBR/MC, and NBR/BHT composite cells to evaluate the MSD of antioxidants (denoted as AO) in NBR matrices. Each NBR molecular chain contained 100 repeating units with an acrylonitrile-to-butadiene ratio of 1:4. The pure NBR unit cell consisted of 4 NBR chains, while AO cells contained 30 antioxidant molecules. Composite NBR/AO cells included 4 NBR chains and 10 AO molecules.

The QM simulations in this study were implemented using the DMol^3^ module of Materials Studio 2020. The specific settings are as follows: The electronic structure calculation is based on the Perdew–Burke–Ernzerhof (PBE) functional of generalized gradient approximation (GGA), combined with the dual-numerical polarization basis set (DNP+) to describe the system wave function. The characteristic of this basis set is that it contains the polarization *p* functions of all hydrogen atoms and has high accuracy. The Semi-core Pseudopotentials method was selected for external processing. The spin multiplicity is set according to the electronic characteristics of the system: For a system containing single-electron free radicals, the spin multiplicity = the number of lone pairs of electrons +1. To improve the convergence of SCF, the Fermi–Amaldi hybrid algorithm (with an initial hybrid coefficient of 0.2) was adopted, and the Fermi level smoothing parameter (smearing width = 0.005 eV) was set. The convergence criteria for geometric optimization were set as energy change <1 × 10^−5^ Hartree and maximum atomic displacement <0.001 A, and the structure was optimized through the BFGS algorithm. The frequency analysis was carried out using the Vibrational Analysis command in the Tools tool to ensure that only one virtual frequency occurs in the transition state structure and the vibration mode is correct.

For MD simulations, the COMPASSⅡ all-atom force field [21], validated for polymer systems, was employed with 50 annealing cycles (200 K → 500 K) to eliminate initial conformation bias. The equilibration protocol consisted of two phases: 200 ps pre-equilibration under the NVT ensemble (298 K, Nosé-Hoover thermostat, 1 ps relaxation time), followed by 500 ps equilibration under the NPT ensemble (298 K, 0.1 MPa, Berendsen barostat, compressibility 4.5 × 10^−5^ bar^−1^) to achieve energy convergence (fluctuation < 0.5 kcal/mol/atom). The final 480 ps trajectory from the production phase was used to calculate solubility parameters via the Hildebrand equation and to analyze molecular mobility through mean squared displacement (MSD) [9,13,16,22,23].

## 3. Results and Discussion

### 3.1. FTIR Analysis

**Figure 2:**
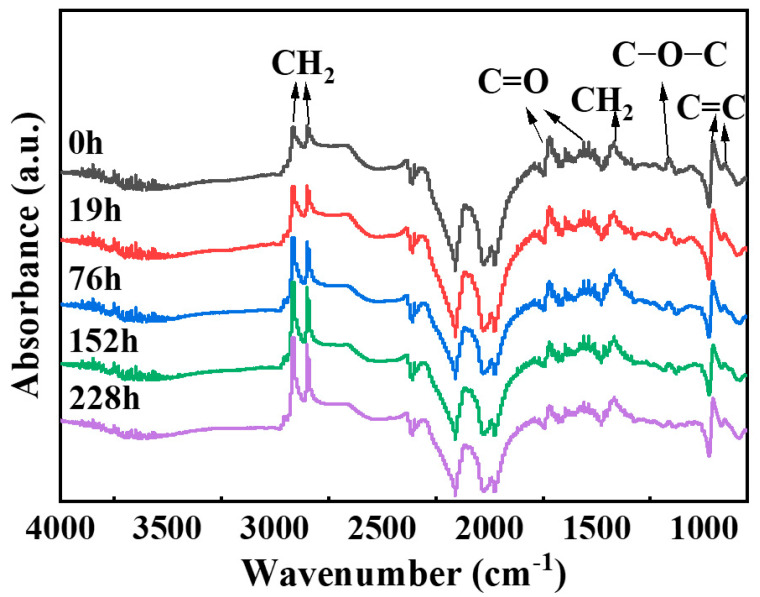
Infrared spectrum changes of nitrile butadiene rubber during aging.

FTIR is a powerful analytical tool that can be used to evaluate the changes in the chemical structure of NBR during the aging process. By comparing the FTIR spectra at different aging stages, characteristic changes related to oxidative aging can be identified. Figure 2 shows the Fourier–transform infrared spectra of NBR before and after thermo-oxidative aging. In Figure 2, the peak at 3365 cm^−1^ corresponds to O-H stretching vibration, while those at 2916 cm^−1^, 2850 cm^−1^, and 1418 cm^−1^ are caused by the asymmetric stretching vibrations, symmetric stretching, and bending vibration of C–H in -CH_2_ [24,25]. The peaks at 1731 cm^−1^, 1540 cm^−1^, and 1162 cm^−1^ arise from C=O stretching and C-O-C stretching vibrations [24,26], and those at 958 cm^−1^ and 903 cm^−1^ are assigned to the vibrations of C-H adjacent to unsaturated bonds in trans-1,4-polybutadiene and vinyl-1,2-polybutadiene units [26,27,28].

Analysis of the intensity changes of each functional group was conducted by examining the peak areas of their respective vibration bands: -CH_2_ (2945–2805 cm^−1^), -C=O (1745–1675 cm^−1^), C=C (976–847 cm^−1^), and C-O-C (1190–1130 cm^−1^). The peak areas are listed in Table 1. There are a large number of methylene groups (-CH_2_) in NBR. Theoretically, the change of methylene groups during the aging process is relatively small [29]. However, due to the crosslinking and degradation of NBR caused by aging, which leads to changes in the surface chemical environment, the relative intensity of the -CH₂ absorption peak is enhanced. In this paper, taking the intensity of the -CH₂ absorption peak as a standard, the changes in the intensities of each absorption peak relative to that of the -CH₂ absorption peak are shown in Table 2. The results show that the intensities of the absorption peaks of C=C, -C=O, and C-O-C all decrease during the aging process [3,30]. This is because during the aging process of NBR materials, the C=C and C=O groups participate in crosslinking reactions, leading to a reduction in their associated infrared absorption peaks as the degree of aging increases [26].

### 3.2. Crosslinking Density Change

**Figure 3:**
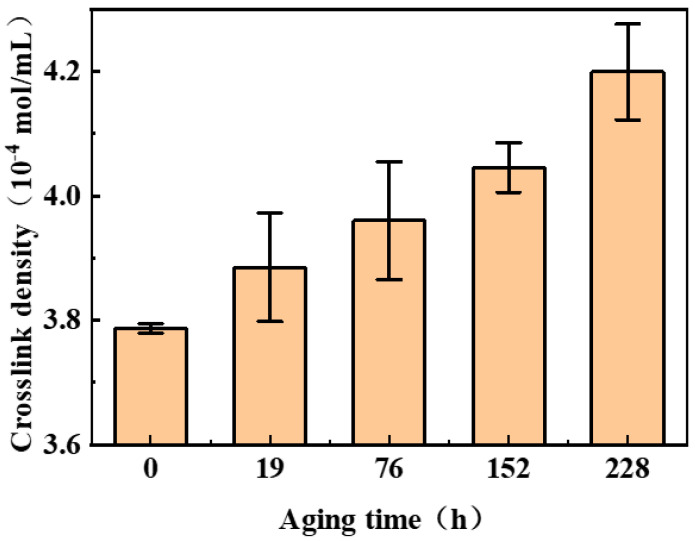
Variation in NBR crosslinking density with aging time.

The crosslinking density of nitrile rubber during the aging process was measured using the equilibrium swelling method. The evolution of NBR’s crosslink density during aging is shown in Figure 3. As observed from the figure, crosslinking dominates the aging process, with crosslink density increasing progressively with aging time—a trend consistent with the previous literature [2,3].

### 3.3. Contact Angle Analysis

Contact angle variations reflect changes in NBR’s surface polarity: more polar functional groups correspond to smaller water contact angles, while fewer polar groups result in larger angles. In NBR, -CH_2_ groups exhibit strong hydrophobicity, whereas oxygen-containing groups like -C=O and C-O-C are highly polar.

**Figure 4:**
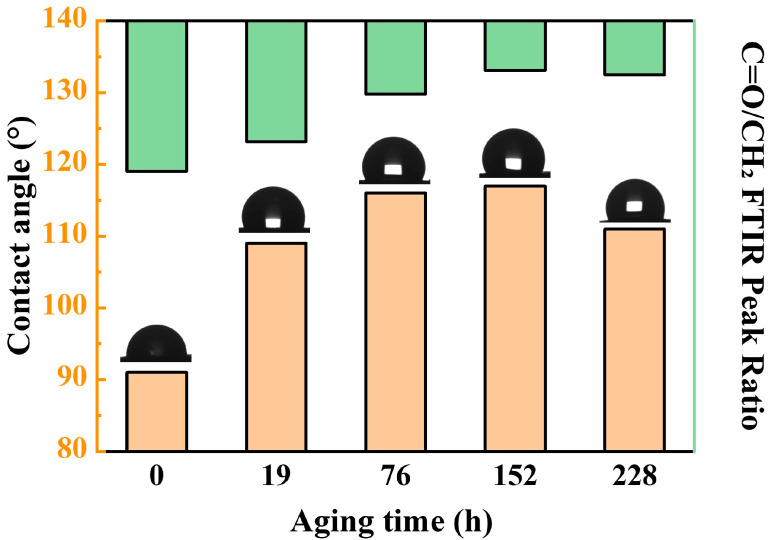
Change in the surface contact angle of NBR during the aging process.

The contact angle evolution of NBR during aging is shown in Figure 4. The results show that the contact angle of NBR first increases and then decreases with the extension of time. Moreover, the increase in the contact angle (enhanced hydrophobicity) was significantly correlated with the decrease in the relative content of C=O, indicating that the change in surface chemical composition dominated the wettability evolution.

### 3.4. Molecular Dynamics Analysis of the Aging Mechanism

In previous studies, changes in surface chemical groups and polarity of NBR during aging were characterized using FTIR and contact angle measurements. This section investigates the underlying mechanisms of these chemical changes through MD simulations, which reveal reaction sites and pathways in NBR molecular chains during thermo-oxidative aging.

NBR is synthesized from butadiene and acrylonitrile monomers with added fillers and antioxidants. This analysis focuses solely on the NBR matrix, excluding additive effects on aging. First, a typical structural unit of NBR was constructed using Materials Studio. QM simulations were then performed to calculate the BDE of NBR’s representative molecular structures, followed by transition state barrier analysis for further decomposition and crosslinking of oxidation products. These computations provide mechanistic insights into NBR aging processes.

#### 3.4.1. Bond Dissociation Energy

**Figure 5:**
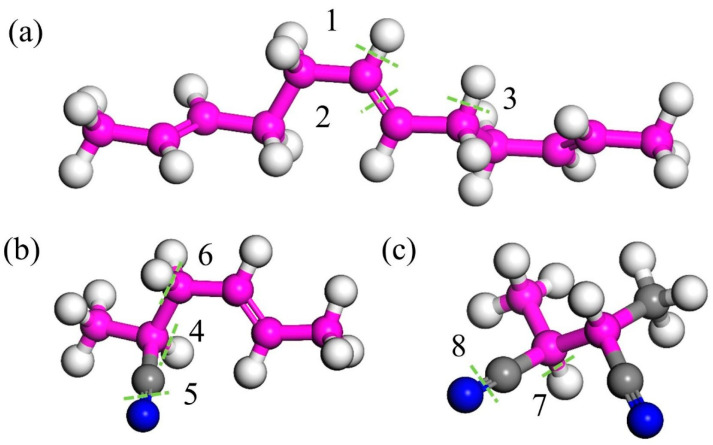
The solution of the typical structure of butylenitrile rubber: (**a**) the butadiene representative unit, (**b**) the acrylonitrile representative unit, and (**c**) acrylonitrile representative unit 2. Where pink beads represent carbon atoms on the carbon skeleton, gray beads represent carbon atoms on the branches, white beads represent hydrogen atoms, and blue beads represent nitrogen atoms. The green dashed line represents the location of key breakage, and its key energy corresponds one-to-one with positions 1–8 in Table 3.

The typical structures of butadiene and acrylonitrile monomers in NBR were analyzed, and the BDE of these typical structures was calculated. The positions of the bonds for which the dissociation energies were calculated are shown in Figure 5, and the calculated dissociation energies are summarized in Table 3.

**Figure 6:**
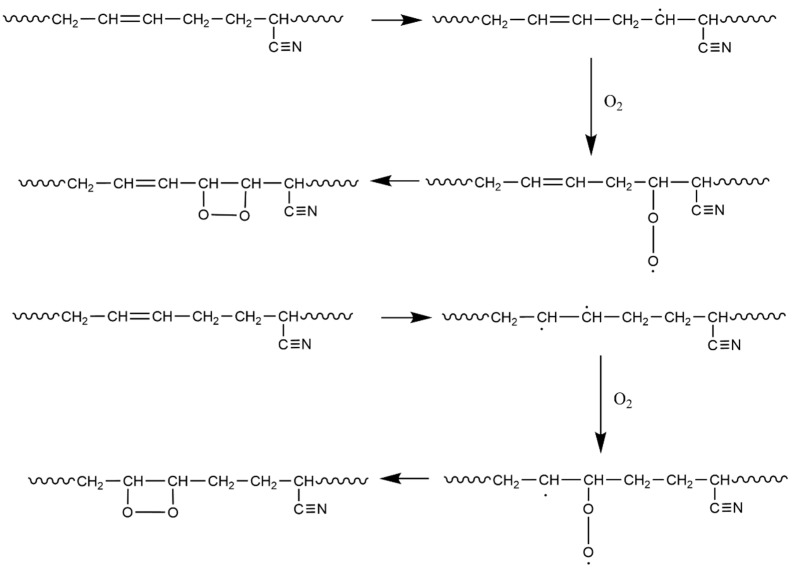
Reaction mechanism of α-H and double bonds with O_2_.

From the table, it can be seen that the C=C double bond (Structure 2 in Figure 5) and C≡N triple bonds (Structures 5 and 8 in Figure 5) exhibit low bond dissociation energies (BDEs). Additionally, the C≡N and C=N bonds in Structure 4c also have low cleavage energies, indicating that these structures are preferentially attacked by O_2_ during aging—a finding consistent with the reduced peak intensities of C=C and C≡N bonds observed in FTIR spectra. We also observed that α-hydrogens adjacent to functional groups (Structures 3, 4, and 6) possess low BDEs. During aging, allylic structures like Structure 3 retain high stability and persist for extended durations [31], making α-hydrogens highly reactive and prone to oxidation. As shown in Figure 6, during oxidation, α-hydrogens or C=C double bonds are activated by environmental factors to form radicals, which react with oxygen to generate new radicals and eventually epoxy structures [9,23].

#### 3.4.2. Reaction Energy Barriers

The epoxy species formed during NBR oxidation are unstable and further react to generate C=O or -OH groups. This section reports transition state barrier calculations for epoxy decomposition into C=O and -OH via reaction pathway searches.

In physical chemistry, the occurrence of chemical reactions should not only consider thermodynamics but also dynamics. The reaction energy barrier reflects the reaction rate. Generally speaking, the lower the reaction energy barrier, the faster the chemical reaction rate. In order to further analyze the oxidation process of NBR, we simulated the E_barrier_ of the NBR reaction with oxygen by QM. In the calculation process, we use the structure in Figure 5b as the representative unit, which contains both C≡N, C=C, and α-H, and the corresponding bond energy is low, which can affect the reaction process of NBR and oxygen.

**Figure 7:**
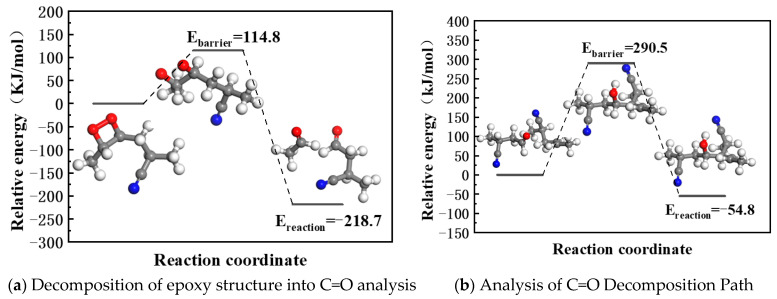
Relationship between energy and chemical structure.

Reaction pathways for epoxy decomposition into C=O and -OH (Figure 6) were analyzed. The calculation results (Figure 7a,b) reveal that epoxy groups do not directly form -OH but proceed through a C=O intermediate. The formation of C=O is accompanied by main chain scission, reducing crosslink density. This conclusion is consistent with the degradation pathway of the anti-degradation agent 6PPD reported by Rossomme, where an epoxy intermediate converts into aromatic compounds (aniline and catechol) and carbonyl products (aldehydes and ketones) [23]. Zheng et al. [32] and Ozonation et al. [33] also reported similar behavior, wherein the O-O bond in the epoxy intermediate state is unstable under thermodynamic or ultraviolet conditions, breaks and decomposes into carbonyl products, and leads to chain opening. These findings support the plausibility of our proposed reaction pathway.

Our calculations also show (Figure 7b) that the oxidized C=O group can react with the unstable hydrogen on the NBR chain to form -OH, triggering crosslinking and spreading aging (with an activation energy of −54.8 kJ/mol). The crosslinking reaction pathway of C=O was confirmed by our experimental evidence and relevant literature. Our infrared spectroscopy results show a significant decrease in the C=O peak intensity during aging (Figure 2), accompanied by an increase in contact angle (Figure 4), indicating reduced surface polarity. This trend corroborates Luo et al.’s [26] conclusion that C=O groups participate in crosslinking reactions during aging. Together, these experimental observations and theoretical calculations provide multi-faceted support for our conclusions.

### 3.5. Antioxidant Screening

This section evaluates the BDEs, solubility characteristics, and migration/diffusion behaviors of four common AOs—4010NA, 4010, MC, and BHT—using MD simulations. Specifically, BDEs serve as critical indicators of radical-trapping efficiency, directly influencing antioxidant performance; solubility parameters reflect the uniformity of AO dispersion in rubber matrices; and diffusion coefficients correlate with AO durability and extraction resistance.

#### 3.5.1. The Free Energy of Dissociation

**Figure 8:**
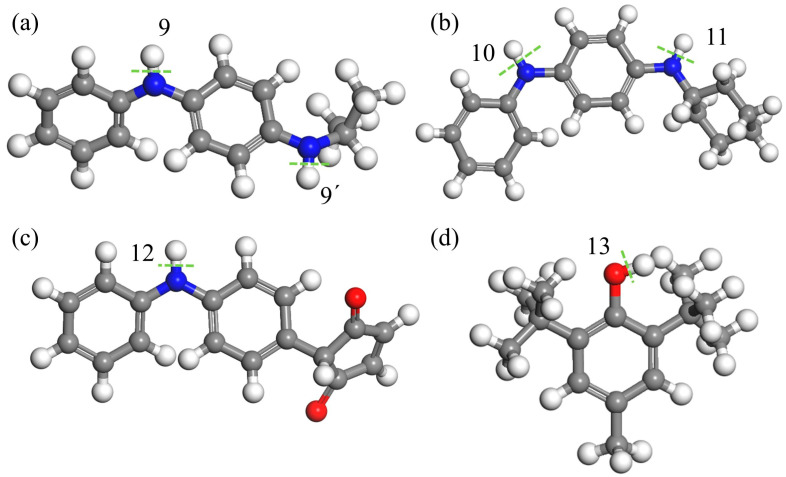
Bond dissociation energies of labile hydrogens: (**a**) 4010NA, (**b**) 4010, (**c**) MC, and (**d**) BHT molecular structure diagram, in which the underlined part is the active hydrogen site. Where red beads represent carbon atoms on the carbon skeleton, gray beads represent carbon atoms on the branches, white beads represent hydrogen atoms, and blue beads represent nitrogen atoms. The green dashed line represents the location of key breakage, and its key energy corresponds one-to-one with positions 9–13 in Table 3.

As analyzed in Section 3.4, isolated double bonds, nitrile groups, and α-H in rubber macromolecules exhibit low bond dissociation energies (BDEs), making them prone to radical generation under thermo-oxidative conditions. Antioxidants (AOs) suppress oxidation by scavenging radicals, thereby delaying aging. The BDE of labile hydrogens in AOs is a critical metric for evaluating antioxidant efficacy. MD calculations of the labile hydrogen BDEs in Figure 8 (summarized in Table 3) show that all AO BDEs exceed those of double bonds and nitrile groups, indicating that AOs cannot protect these structures. AO BDEs (346.3–377.3 kJ/mol) closely match α-H BDE (371.4 kJ/mol), enabling partial protection against α-H decomposition. Among the tested AOs, BHT has the lowest BDE (346.3 kJ/mol) and the best protective effect.

#### 3.5.2. Solubility Parameters

The solubility parameters of each structure were obtained by statistically analyzing the equilibrium structure according to the MD calculation process in Section 2.3 (taking the average of the three segments). Solubility parameters of AOs and their solubility differences (Δδ) with NBR are listed in Table 4. Solubility parameters evaluate the compatibility of blended materials. According to semi-empirical methods, materials are fully miscible when Δδ < 2.05 MPa^1^/^2^ and partially miscible when Δδ ranges from 2.05 MPa^1^/^2^ to 10.02 MPa^1^/^2^ [9]. In Table 4, the solubility order of AOs in NBR is as follows: BHT > 4010 > 4010 Na > MC. However, all AOs can partially dissolve in NBR and serve as antioxidants for NBR.

#### 3.5.3. Mean Square Displacement

Based on the AOs/NBR composite cell model constructed in Section 2.3, the composite cells were annealed and pre-balanced according to MD, and the balanced cells were statistically analyzed. And the MSD and diffusion coefficient are used to characterize the anti-extraction property of AO in the rubber matrix, which is expressed as follows [26,34]:

(4) $$MSD(t)=\frac{1}{N}\sum_{i=1}^{N}\langle {\left|r_{i}(t+t_{0})-r_{i}(t_{0})\right|}^{2}\rangle$$

(5) $$D=\frac{1}{6N}\underset{x\to \infty }{\lim}\frac{d}{dt}\sum_{i=1}^{N}\langle {\left|r_{i}(t+t_{0})-r_{i}(t_{0})\right|}^{2}\rangle =\frac{1}{6}\underset{x\to \infty }{\lim}\frac{d}{dt}MSD(t)$$

where *N* is the number of molecules, *D* is the diffusion coefficient, $r_{i}(t_{0})$ is the displacement at $t_{0}$, and $r_{i}(t+t_{0})$ is the displacement at $t+t_{0}$. The value of the diffusion coefficient in the text is equal to 1/6 of the slope of the *MSD* linear region.

**Figure 9:**
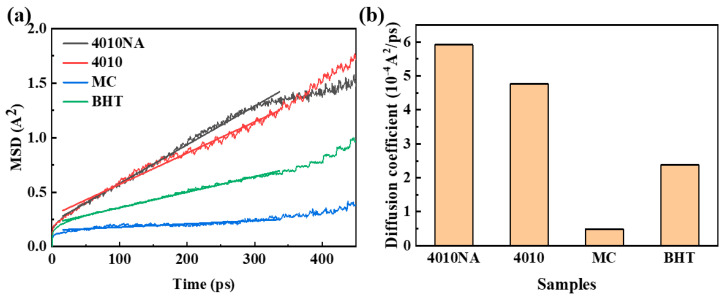
(**a**) MSD curves and (**b**) diffusion coefficient of 4 selected antioxidants in the NBR matrix.

The MSD curves and diffusion coefficients of four antioxidants in NBR are shown in Figure 9. Analysis shows that MC has the lowest migration rate in NBR, followed by BHT, and 4010 and 4010NA have poor anti-migration ability in NBR.

#### 3.5.4. Summary

Table 5 comprehensively summarizes the performance evaluations of four antioxidants, namely 4010NA, 4010, MC, and BHT, in terms of free radical scavenging ability, solubility, and diffusion coefficient within nitrile–butadiene rubber (NBR). The results indicate that MC exhibits outstanding anti-migration properties, maintaining a relatively stable distribution within the NBR matrix. However, it shows poor free radical scavenging efficiency and limited compatibility with NBR, potentially compromising its antioxidant effectiveness in practical applications. Due to their similar molecular structures, 4010NA and 4010 demonstrate comparable performance in free radical scavenging, solubility, and diffusion coefficient in NBR. Notably, 4010 outperforms 4010NA across all indicators, presenting a more balanced and efficient antioxidant profile. Among these antioxidants, BHT stands out with its superior free radical scavenging ability, excellent solubility, and good anti-migration properties. Its comprehensive performance makes it the most optimal antioxidant choice for NBR systems.

## 4. Conclusions

This study systematically reveals the thermo-oxidative aging mechanism of NBR under thermo-mechanical coupling environments through a multi-scale research approach combining experimental characterization and MD simulations. A multi-dimensional evaluation framework for antioxidants was established, providing an important theoretical basis for the development of high-performance rubber materials.

Our results showed that absorption peaks of C=C and C=O groups decreased during aging, corroborated by quantum mechanics (QM) simulations confirming preferential oxidation of α-H, C=C, and C≡N groups in NBR. Crosslink density measurements revealed increased crosslinking after aging, indicating dominant chain reorganization during oxidation.

Multidimensional evaluation results from molecular dynamics simulations of antioxidants demonstrate that BHT exhibits the most outstanding comprehensive performance among the four candidate antioxidants. In terms of free radical scavenging ability, BHT has an extremely low OH bond dissociation energy (BDE) of 346.3 kJ/mol, indicating that it can efficiently quench free radicals by releasing hydrogen atoms with minimal energy consumption. Regarding compatibility with the nitrile–butadiene rubber (NBR) matrix, BHT shows a solubility parameter difference (Δδ) of only 2.95 with NBR, significantly lower than that of other antioxidants. This suggests strong intermolecular forces between BHT and NBR, enabling uniform dispersion within the rubber matrix. Additionally, the simulation results confirm that BHT has a moderate diffusion rate within NBR, endowing it with excellent anti-migration properties and effectively preventing performance degradation caused by antioxidant loss due to migration. Through quantitative analysis of free radical scavenging efficiency, compatibility, and anti-migration characteristics, BHT is identified as the optimal choice among the four antioxidants, providing an ideal solution for enhancing the antioxidant performance of NBR materials.

This research provides a multi-faceted analysis of NBR’s thermo-mechanical aging mechanism, deepening understanding of rubber degradation under coupled environments. Additionally, it establishes a multi-dimensional evaluation method for antioxidants in NBR, offering theoretical guidance for preparing high-performance antioxidant NBR materials.

## Figures and Tables

**Table 1:** Changes in characteristic peaks during the aging process of nitrile butadiene rubber.

Aging Time	CH_2_	C=O (1731)	C=C (958)	C-O-C (1162)
0 h	3.41	2.40	7.19	0.82
19 h	3.95	2.20	7.46	0.77
76 h	4.81	1.63	5.59	0.61
152 h	5.50	1.25	5.30	0.50
228 h	5.52	1.37	6.03	0.27

**Table 2:** Change in characteristic peak strength relative to methylene peak during the aging of NBR.

Aging Time	CH_2_	C=O	C=C	C-O-C
0 h	1.00	0.70	2.11	0.24
19 h	1.00	0.56	1.89	0.19
76 h	1.00	0.34	1.16	0.13
152 h	1.00	0.23	0.96	0.09
228 h	1.00	0.25	1.09	0.05

**Table 3:** Energy and corresponding bond dissociation energies for different structures.

Structural Units	Dissociation Energy (kJ/mol)
H·	-
Butadiene Representative Unit	-
Acrylonitrile Representative Unit	-
Acrylonitrile Representative Unit 2	-
Position 1	467.1
Position 2	269.2
Position 3	371.4
Position 4	377.1
Position 5 (generates C≡N)	266.6
Position 5-2 (generates C−N)	438.9
Position 6	370.4
Position 7	390.7
Position 8 (generates C≡N)	314.0
Position 8-2 (generates C−N)	276.7
Position 9/9’	353.2/380.7
Position 10	351.4
Position 11	372.6
Position 12	377.3
Position 13	346.3

**Table 4:** Solubility parameters of AOs and their solubility differences (Δδ) with NBR.

Structure	NBR	4010NA	4010	MC	BHT
Solubility parameter δ	31.60 ± 0.07	38.03 ± 0.12	35.01 ± 0.13	39.09 ± 0.10	28.65 ± 0.11
△δ	-	6.43	3.41	7.49	2.95

**Table 5:** Summary of free radical trapping ability, compatibility, and migration of four AOs in nitrile rubber.

Structure	4010NA	4010	MC	BHT
Free radical scavenging capacity	3 (353.2)	2 (351.4)	4 (377.3)	1 (346.3)
△δ	3 (6.43)	2 (3.41)	4 (7.49)	1 (2.95)
Diffusion capacity	4	3	1	2

## Data Availability

The original contributions presented in this study are included in the article. Further inquiries can be directed to the corresponding authors.
